# Skin Conductance Reactivity as a Predictor of Stroke-Induced Posttraumatic Stress Disorder Symptoms: A Dimensional Approach

**DOI:** 10.1155/2023/6671337

**Published:** 2023-07-15

**Authors:** Corinne Meinhausen, Gabriel J. Sanchez, Donald Edmondson, Ian M. Kronish, Joseph E. Schwartz, Rebecca Hinrichs, Tanja Jovanovic, Jennifer A. Sumner

**Affiliations:** ^1^Department of Psychology, University of California, Los Angeles, Los Angeles, CA, USA; ^2^Center for Behavioral Cardiovascular Health Columbia University Irving Medical Center, New York, NY, USA; ^3^Department of Psychology, St. John's University, Queens, NY, USA; ^4^Department of Psychiatry and Behavioral Health, Renaissance School of Medicine, Stony Brook University, Stony Brook, NY, USA; ^5^Department of Psychiatry and Behavioral Sciences, Emory University, School of Medicine, Atlanta, GA, USA; ^6^Department of Psychiatry and Behavioral Neurosciences, Wayne State University, Detroit, MI, USA

## Abstract

**Background:**

Posttraumatic stress disorder (PTSD) symptoms can develop following acute, life-threatening medical events. This study explores a potential biomarker of PTSD risk that is novel to a medical trauma population: a noninvasive, mobile skin conductance (SC) measurement.

**Methods:**

Participants (*n* = 64) were enrolled inhospital following a stroke or transient ischemic attack (TIA). Mobile measurement of SC reactivity to recalling the stroke/TIA traumatic event was conducted at hospital bedside in the days following the stroke/TIA. PTSD symptoms that developed in response to the stroke/TIA were measured at 1-month follow-up. We tested the association between SC reactivity and total 1-month PTSD symptoms, as well as PTSD symptom dimensions of fear and dysphoria.

**Results:**

In unadjusted analyses, there were significant positive associations between inhospital SC reactivity to recalling the stroke/TIA traumatic event and higher-order fear-related symptoms (*r* = .30, *p* = .016), as well as lower-order fear-related symptoms of anxious arousal (*r* = .27, *p* = .035) and avoidance (*r* = .25, *p* = .043) at 1 month. Associations between SC reactivity and the fear, anxious arousal, and avoidance symptom dimensions remained significant in multivariable regression models that adjusted for relevant covariates including age, gender, stroke severity, medical comorbidity, and psychosocial factors. Although there was a positive association observed between SC reactivity to recalling the stroke/TIA event and total PTSD symptom severity at 1-month follow-up, it did not reach the level of statistical significance (*r* = .23, *p* = .070). Further, no significant association was detected for dysphoria-related symptoms (*r* = .11, *p* = .393).

**Conclusions:**

This is the first study to test the prospective association of SC reactivity with PTSD symptom development following a medical trauma. The findings indicate that mobile measures of SC reactivity may be useful for inhospital identification of individuals at risk for fear-related PTSD symptom development following a medical event and highlight the potential mechanisms involved in the development of these symptoms following a medical event.

## 1. Introduction

Strokes are highly prevalent, life-threatening medical emergencies and a leading cause of death and disability globally [1]. Strokes occur when blood flow to an area of the brain is interrupted, and this may cause damage or death to surrounding brain cells. Transient ischemic attacks (TIAs), though previously conceptualized as benign episodes of temporary (<24 hours) stroke-like symptoms, are increasingly recognized as clinically significant events that may also result in long-lasting brain injury [2]. Symptoms of a stroke (e.g., numbness, paralysis of limbs, and aphasia) can be terrifying and trigger significant psychological distress [3]. In fact, stroke and TIA patients—as well as their caregivers—often cite chronic emotional distress following the event as one of the most impactful challenges faced during recovery [4].

Although posttraumatic stress disorder (PTSD) was originally conceptualized as developing in response to discrete and external traumatic events (e.g., assault, combat), a growing body of evidence has demonstrated that acute medical events like strokes and TIAs can also trigger the development of PTSD symptoms (medically induced PTSD; [5, 6]). Meta-analytic evidence suggests that nearly one in four stroke/TIA survivors will develop elevated PTSD symptoms during the first year following their event [6]. Individuals who develop stroke-induced PTSD symptoms also have poorer physical recovery, including a heightened risk of stroke recurrence and disability, in addition to adverse psychological outcomes [7, 8].

### 1.1. Psychophysiological Reactivity and PTSD Risk

Despite the prevalence of stroke-induced PTSD symptoms observed in patients, screening procedures for risk have yet to be implemented [9]. Timely identification of those individuals at risk of developing PTSD symptoms may inform prevention efforts to promote patients' mental and physical recovery after stroke/TIA. Heightened psychophysiological reactivity to trauma-related stimuli as measured by skin conductance (SC) provides objective measurement of sympathetic nervous system response to trauma reminders [10] and may be a biomarker for heightened risk of PTSD symptom development. An inhospital study that used mobile assessments of SC reactivity measured during the acute aftermath of nonmedical trauma exposure found that heightened SC reactivity during a standardized trauma interview predicted subsequent development of PTSD symptoms and more chronic PTSD symptom manifestations [11]. Another recent study found that poststroke heightened SC was associated with several medically induced PTSD risk factors, including greater cumulative trauma burden and inhospital benzodiazepine administration [12]. Although SC measurement has traditionally been conducted in a research setting among individuals exposed to external (nonmedical) trauma, recently developed mobile SC assessments are a low-cost, noninvasive alternative, offering naturalistic measurement while patients are under the care of medical professionals following a serious medical event [11]. However, research exploring objective risk factors for PTSD symptom development like SC reactivity in individuals following a medical trauma is lacking.

### 1.2. Dimensional Analysis of PTSD Symptoms

PTSD is a remarkably heterogeneous disorder; a *DSM-5* PTSD diagnosis can potentially occur as the result of 636,120 potential combinations of individual symptoms [13, 14]. Confirmatory factor analysis has been used to identify distinct underlying dimensions of PTSD [15, 16], and researchers have increasingly advocated for investigating PTSD not solely as a unitary phenotype, but as a constellation of symptom dimensions that may have differential associations with intermediary disease processes [17]. Research that utilizes more nuanced measurement of underlying symptom dimensions can then be used to inform more targeted intervention efforts.

The symptoms of PTSD broadly fall into two distinct categories: fear and dysphoria [17]. Fear is considered an alarm response to real or perceived threatening stimuli, whereas dysphoria denotes the absence of positive mood and difficulty experiencing pleasure. These higher-order symptom dimensions of fear and dysphoria can be decomposed into lower-order symptom dimensions, and the five-factor dysphoric arousal model is a well-supported representation of the latent structure of PTSD based on *DSM-IV* and *DSM-5* criteria [15, 18]. Lower-order fear-related PTSD symptoms include symptoms related to reexperiencing the traumatic event (e.g., flashbacks or intrusive thoughts about the traumatic event), active avoidance (e.g., avoiding external trauma reminders and memories of the traumatic event), and anxious arousal (e.g., hypervigilance, startling easily). In contrast, dysphoria-related PTSD symptoms are composed of two lower-order dimensions including dysphoric arousal (e.g., difficulty concentrating, irritability) and emotional numbing (e.g., loss of interest, detachment from others). Dysregulated fear responding, as demonstrated by elevated psychophysiological responses during experimental fear conditioning and extinction paradigms, is thought to be involved in the development and maintenance of PTSD [19–24]. Further, fear-related PTSD symptoms entail heightened reactivity and, as such, may be detectable by measurements that capture changes in physiological arousal such as SC. If so, then posttrauma SC reactivity could be particularly relevant to the subsequent development of fear-related PTSD symptoms [11, 25–27].

### 1.3. Aims of the Current Study

The current study employed a longitudinal design to examine the extent to which SC reactivity while recalling a stroke/TIA traumatic event—measured inhospital—was related to stroke-induced PTSD symptoms one month later. Given the heterogeneity of PTSD, in addition to predicting total PTSD symptoms at 1-month follow-up, we utilized a dimensional approach to PTSD symptom analysis, examining higher-order dimensions of fear and dysphoria and the five lower-order symptom dimensions of the dysphoric arousal model. We hypothesized that greater SC reactivity while recalling the stroke/TIA event would predict higher 1-month total stroke-induced PTSD symptom levels, as in research with nonmedical trauma-exposed individuals (e.g., [11]). Additionally, given that SC reactivity reflects sympathetic activity linked to physiological arousal, we hypothesized that greater SC reactivity would be particularly related to higher fear-related PTSD symptoms.

## 2. Methods

### 2.1. Participants

Participants were evaluated for stroke or TIA at the NewYork-Presbyterian/Columbia University Irving Medical Center and were subsequently enrolled during hospitalization in the Reactions to Acute Care and Hospitalization for Suspected Stroke (REACH Stroke) study. REACH Stroke is a prospective longitudinal observational cohort study examining risk factors for, and outcomes of, PTSD symptom development following a stroke or TIA. Study enrollment began in 2015, and criteria for enrollment included fluent in English or Spanish, ≥18 years of age, and a diagnosis of suspected stroke or TIA by the treating neurologist. Exclusion criteria included the following: severe stroke symptoms according to the National Institutes of Health (NIH) Stroke Scale (score > 14 [28]); significant aphasia, dysarthria, or cognitive impairment; severe mental illness; terminal noncardiac medical comorbidities; and unavailability for follow-up visits. All eligible participants were provided with study detail and gave written informed consent prior to their participation. This study was approved by the Columbia University Irving Medical Center Institutional Review Board.

### 2.2. Procedure

Participant's sociodemographic information was collected during study enrollment; information regarding medical characteristics (e.g., prior stroke, medical comorbidity) and stroke/TIA severity were obtained from participants' medical records. During hospitalization, trained research assistants assessed several relevant psychosocial factors (described below). Additionally, a subset of the participants enrolled in the REACH Stroke study were invited to complete an inhospital psychophysiology substudy (i.e., the PhenX Toolkit Baseline and Trauma Challenge Psychophysiology Recording protocol; for details about this protocol, see https://www.phenxtoolkit.org/protocols/view/630901). Data for the psychophysiology substudy were collected between August 2017 and June 2019. The PhenX protocol is brief (<15 minutes), consisting of a 2-minute resting baseline and a standardized trauma interview (15 questions related to the experience of the traumatic event; here, the stroke/TIA event). SC response was measured continuously during the resting baseline and trauma interview. Approximately one month after enrollment in the study, PTSD symptoms that developed in response to the stroke/TIA event were measured via phone interviews. All assessments were conducted in English or Spanish, based on participant preference.

### 2.3. Measures

#### 2.3.1. Sociodemographics

Participants reported their age, gender (male, female), race/ethnicity (Hispanic, non-Hispanic Black, non-Hispanic White, and other), and educational attainment (less than high school/some high school, high school degree, trade school/some college, college graduate, and graduate school).

#### 2.3.2. Medical Characteristics

Several medical characteristics were extracted from information in participants' medical charts. For example, study neurologists categorized the index event as stroke, TIA, or other, and stroke severity was quantified using the 11-item NIH stroke scale [28], and prior history of stroke/TIA (dichotomized as present/absent) was documented. Information about history of a variety of medical conditions was also extracted from medical charts and integrated to calculate the Charlson Comorbidity Index, a 17-item measure of medical comorbidity [29].

#### 2.3.3. Psychosocial Risk Factors

During the index hospitalization, a 7-item measure of perceived threat experienced during the stroke/TIA event was administered; responses were rated on a 4-point scale (1=not at all, 4=extremely) and were summed (Cronbach's *α* = .88; [30]). Acute posttraumatic stress symptoms due to the stroke/TIA event were also rated on a 5-point scale (1=not at all, 5=extremely) using a 14-item version of the Acute Stress Disorder Scale and were summed to create a total score (Cronbach's *α* = .77; [31]).

#### 2.3.4. SC Reactivity to Recalling the Stroke/TIA Event

SC measurement was performed by trained research coordinators at bedside during hospitalization. Electrodes were attached with Velcro to the nondominant intermediate phalanges of the participant's index and middle finger. Isotonic paste was applied to electrodes prior to data acquisition to enhance signal quality and skin contact. A sampling rate of 10 Hz was used during data acquisition, which consisted of a 2-minute resting baseline period followed by a standardized trauma interview in which SC reactivity was measured using the eSense SC system (Mindfield Biosystems, Inc., Berlin, Germany).

SC reactivity to the trauma interview was calculated by subtracting the average SC level in microSiemens (*μ*S) during the last 30 seconds of the baseline resting period from the maximum SC level (*μ*S) during the trauma interview, as in prior mobile SC research [11, 32]. Greater positive values indicate more psychophysiological reactivity while recalling the stroke/TIA traumatic event (compared to baseline).

#### 2.3.5. Stroke-Induced PTSD Symptoms

Participants were asked about the presence of *DSM-5* PTSD symptoms that onset in response to the stroke/TIA event using the 20-item PTSD Checklist for *DSM-5* (PCL-5; [33]) at 1-month follow-up. Individual responses were rated on a 5-point Likert scale (0=not at all, 4=extremely) with respect to the past month and were summed to create a total PTSD symptom severity score (Cronbach's *α* = .87). In addition, several PTSD symptom dimension scores were calculated, including higher-order dimensions of fear (9-item measure; Cronbach's *α* = .80) and dysphoria (11-item measure; Cronbach's *α* = .79) and the five lower-order dimensions of the dysphoric arousal model (Cronbach's *α* ranging from .31 to .78; lower internal consistency was observed for the 2-item dimensions). Scores ≥ 33 on the PCL-5 have been found to suggest probable PTSD [34], and we presented information on probable PTSD status at 1-month follow-up for descriptive purposes. Although there were too few participants with probable PTSD at 1 month to analyze as a secondary outcome, we focused on continuous PTSD symptoms as our outcome of interest given that even the development of subthreshold PTSD symptoms following trauma exposure can be associated with clinically significant distress and impairment [35].

### 2.4. Data Analysis

Descriptive statistics were calculated for the analytic sample. We also compared individuals who completed the psychophysiology substudy who were and were not included in this analytic sample on demographics. Unadjusted analyses were performed to examine the relations between SC reactivity to recalling the stroke/TIA event and (1) total PTSD symptom severity, (2) higher-order fear and dysphoria symptom dimensions, and (3) lower-order symptom dimensions of reexperiencing, avoidance, anxious arousal, dysphoric arousal, and emotional numbing. In the case of significant unadjusted correlations of SC reactivity with PTSD symptoms, we then performed multivariable linear regression to test whether the associations remained significant when accounting for relevant covariates. We selected the following covariates based on their previously established links with medically induced PTSD risk: age, gender, stroke severity, medical comorbidity, and psychosocial factors including perceived threat during the stroke/TIA and acute posttraumatic stress symptoms [7, 36, 37]. We tested for, and found no evidence of, nonlinear effects of age and thus only included models with linear effects of age.

To maximize the sample size for adjusted analyses, we used imputed scale scores for psychosocial factor covariates (perceived threat and acute posttraumatic stress symptoms) in the relatively few cases where a participant completed some, but not all, items of a scale. For these variables, a three-step strategy was used to impute the scale score. First, the scale's Cronbach's alpha was calculated based on the data for those who answered all items. Second, if *α* ≥ 0.80, the Spearman-Brown formula was used to estimate the minimum number of items, *k*, required for Cronbach's alpha to be ≥0.70; if *α* < 0.80, we determined the minimum number of items required for Cronbach's alpha to not decrease by more than 0.10 (e.g., if *α* for all items was 0.75, we determined the minimum number of items required for *α* to remain ≥0.65; see Supplementary Table 1 for specific values). Last, using the data for all participants with ≥*k* nonmissing items, the expectation-maximization algorithm within the MI procedure of SAS Version 9.4 [38] was used to estimate the expected value of the scale score conditional on the responses to those items that were answered. For those with no missing items, this score was identical to their original scale score. Using imputed scale scores for these two variables allowed us to retain 6 participants, for a total of 56 participants in adjusted models.

We also conducted two sets of sensitivity analyses. First, to assess whether findings that emerged for unadjusted associations of SC reactivity with PTSD symptoms were due to greater psychophysiological reactivity—rather than just higher resting baseline levels of SC—we tested whether baseline SC levels were related to the PTSD symptom variables. Second, we examined whether associations of SC reactivity with PTSD symptoms due to the stroke/TIA event were robust to preexisting PTSD due to a prior trauma given that preexisting PTSD could increase risk of developing new PTSD symptoms in response to the stroke/TIA event. Participants' past-month PTSD symptoms that developed in response to their most distressing prior trauma were assessed with the PCL-5 during the baseline hospitalization, and we repeated the multivariable linear regression models from the main analyses when excluding the 4 individuals with probable PTSD due to a prior trauma (PCL-5 scores ≥ 33).

Analyses were conducted with SPSS Version 27.0 (IBM Corp., Armonk, NY), and *p* < .05 indicated statistical significance.

## 3. Results

### 3.1. Participant Characteristics

The sample comprised individuals with useable SC data and PTSD symptom data at the 1-month follow-up (*n* = 65). One individual had a total PTSD symptom severity score at follow-up that was identified as an extreme outlier using Tukey's test with Tukey's hinge distance factor of 3 and was removed, resulting in a final analytic sample of 64 individuals. We also excluded “non-responder” participants who did not exhibit a psychophysiological response to the trauma interview (*n* = 5; i.e., SC levels were below 1 *μ*S for the duration of the trauma interview), as in prior research [39]. Participants in the analytic sample (*n* = 64) were similar to those who were excluded due to unusable SC data (e.g., recorder malfunction; *n* = 5), SC nonresponder status (*n* = 5), or missing 1-month PTSD symptom data (*n* = 25) across age, gender, race/ethnicity, and educational attainment (Supplementary Table 2).

Characteristics for participants in the analytic sample, including possible and observed ranges for relevant measures, are detailed in Table 1. Mean participant age was approximately 61 years old, and the sample was diverse in regard to gender, race/ethnicity, and educational attainment. The vast majority of individuals (89.1%) were diagnosed with a stroke for the index hospitalization that resulted in their enrollment in the study. Only 18.8% of participants had a history of prior stroke/TIA. Many participants also endorsed perceived threat during hospitalization and symptoms of acute posttraumatic stress in response to the stroke/TIA event.

**Figure 1:**
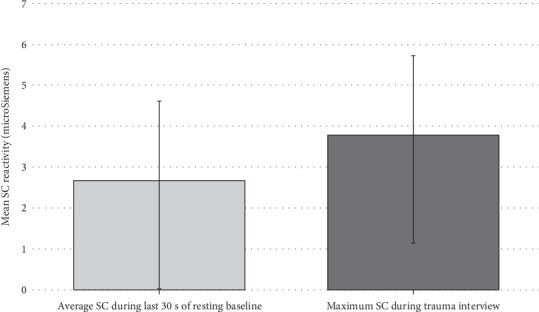
Mean skin conductance (SC) levels (1) during the last 30 seconds of the resting baseline recording period and (2) for the maximum response to recalling the stroke/transient ischemic attack event, *n* = 64. Error bars indicate ±1 standard error (SE).

The average time between study enrollment after stroke/TIA and the psychophysiology substudy was 1.69 days (SD = 1.95, range = 0 − 8). SC during the trauma interview (*M* = 3.77*μ*S, range = 0.77 − 14.94) was significantly greater than the baseline SC level (*M* = 2.67 *μ*S, range = 0.66 − 11.24), *t*(63) = 6.13, *p* < .001 (Figure 1). The mean SC reactivity to recalling the stroke/TIA trauma (max SC during trauma interview minus last 30 seconds of baseline SC) was 1.11 *μ*S, and patients exhibited a broad range of SC responses (range = −0.22 − 6.03 *μ*S).

### 3.2. Inhospital SC Reactivity and PTSD Symptoms after Stroke/TIA

**Figure 2:**
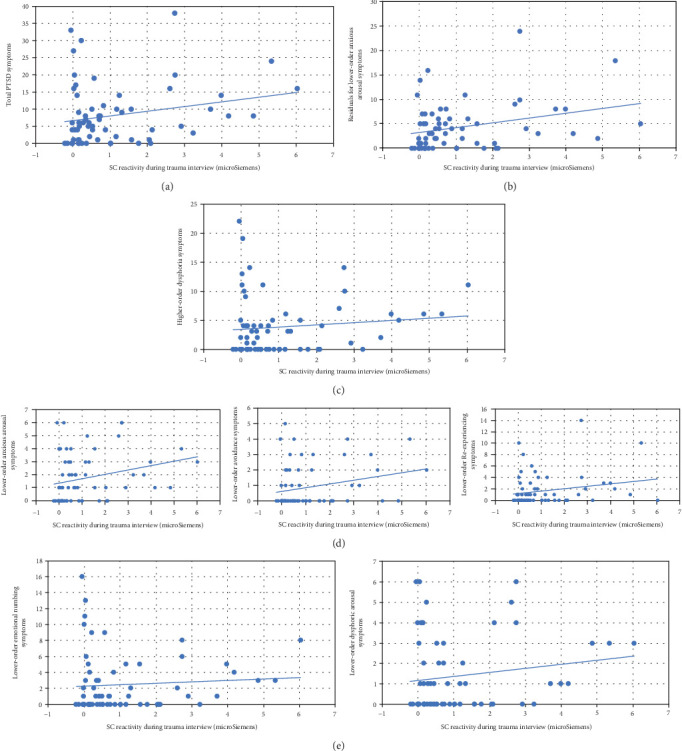
Scatterplots for unadjusted associations between skin conductance (SC) reactivity to recalling the traumatic event and posttraumatic stress disorder (PTSD) symptoms at 1 month that developed in response to the stroke/transient ischemic attack event. Plots display (a) total PTSD symptom severity, (b) higher-order fear symptoms, (c) higher-order dysphoria symptoms, (d) lower-order fear symptom dimensions (anxious arousal, avoidance, and reexperiencing), and (e) lower-order dysphoria symptom dimensions (emotional numbing and dysphoric arousal).

There was a positive association of small-to-medium effect size observed between SC reactivity to recalling the stroke/TIA event and total PTSD symptom severity at 1-month follow-up, but it did not reach the level of statistical significance (*r* = .23, *p* = .070). However, SC reactivity was significantly positively correlated with the higher-order fear dimension, with a medium effect size (*r* = .30, *p* = .016). Of the lower-order fear-related symptom dimensions examined, significant positive associations of a medium size were detected for anxious arousal (*r* = .27, *p* = .035) and avoidance symptoms (*r* = .25, *p* = .043). The correlation between SC reactivity and the other lower-order fear dimension—reexperiencing symptoms—was positive but slightly smaller and not statistically significant (*r* = .22, *p* = .087). Smaller associations were observed for higher-order dysphoria symptoms or its lower-order symptom dimensions, and they did not reach the level of statistical significance (rs < .17, ps > .20; Table 2). Scatterplots for the unadjusted associations of SC reactivity with PTSD symptoms are presented in Figure 2.

**Figure 3:**
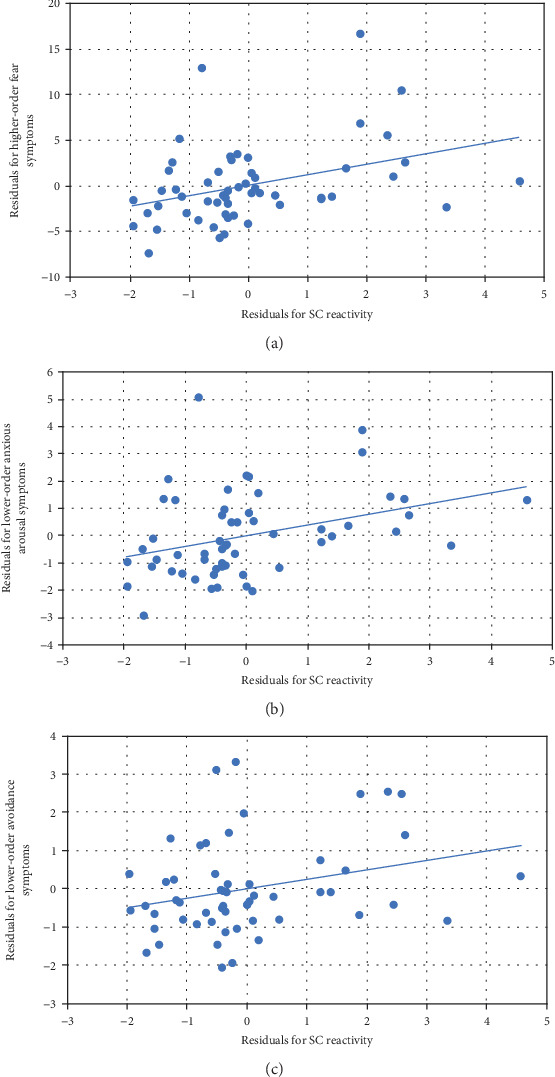
Partial correlations and associated scatterplots of the residuals from the regression predicting skin conductance (SC) reactivity from demographic, medical, and psychosocial covariates and the residuals from the regression predicting posttraumatic stress disorder (PTSD) symptoms examined in adjusted analyses from demographic, medical, and psychosocial covariates. Plots display (a) higher-order fear symptoms (partial *r* = 0.37, *p* < .01), (b) lower-order anxious arousal symptoms (partial *r* = 0.36, *p* = .01), and (c) lower-order avoidance symptoms (partial *r* = 0.28, *p* = .047).

In fully adjusted models that accounted for age, gender, stroke severity, medical comorbidity, and psychosocial risk factors, SC reactivity remained significantly positively associated with higher-order fear symptoms (*β* = .36, *p* = .007) and lower-order anxious arousal (*β* = .32, *p* = .012) and avoidance symptoms (*β* = .27, *p* = .038; Table 3). Although we prioritized the multivariable regression analyses to present effects associated with SC reactivity alongside those for other potential risk factors for PTSD symptoms after stroke/TIA, we also present partial correlations and the corresponding scatterplots for these associations in Figure 3 for comparisons with the unadjusted correlations. Partial correlations accounting for demographics, medical characteristics, and other psychosocial risk factors were slightly larger in size compared to the unadjusted associations.

### 3.3. Sensitivity Analyses

To test whether significant associations between SC reactivity and PTSD symptoms induced by stroke/TIA were driven by an individual's SC response to recalling the trauma rather than their resting baseline SC levels, we performed sensitivity analyses testing the association between resting baseline SC levels (average SC during the last 30 seconds of the baseline recording period) with higher-order fear and lower-order anxious arousal and avoidance symptoms. We found no significant associations between baseline SC level and higher-order fear (*r* = .05, *p* = .67), lower-order anxious arousal (*r* = .12, *p* = .36), or lower-order avoidance PTSD symptoms (*r* = .06, *p* = .65). These findings suggest that the associations observed between SC reactivity and these PTSD symptoms at 1-month follow-up were specific to psychophysiological reactivity to recalling the stroke/TIA traumatic event and not a result of baseline differences in SC levels.

We also examined whether associations of SC reactivity with PTSD symptoms due to the stroke/TIA event were robust to preexisting PTSD due to a prior trauma, given that preexisting PTSD could increase risk of developing new PTSD symptoms in response to the stroke/TIA event. Indeed, there was a significant positive association between past-month PTSD symptoms due to a prior trauma at baseline and stroke/TIA-induced PTSD symptoms at 1-month follow-up (*r* = .58, *p* < .001). When we repeated the multivariable linear regression models from the main analyses when excluding the 4 individuals with probable PTSD due to a prior trauma, SC reactivity remained a significant predictor of higher-order fear, lower-order anxious arousal, and lower-order avoidance PTSD symptoms that developed in response to the stroke/TIA event, and effect sizes were just slightly attenuated (Supplementary Table 3).

## 4. Discussion

We used SC reactivity during a trauma recall interview conducted during hospitalization to test whether an objective measure of psychophysiological reactivity was prospectively associated with stroke/TIA-induced PTSD symptoms assessed 1 month after hospital discharge. Although inhospital SC reactivity to recalling the traumatic event was not significantly associated with total PTSD symptom severity, SC reactivity did prospectively predict fear-related PTSD symptoms. Specifically, greater SC reactivity predicted the development of higher-order symptoms of fear and lower-order symptoms of anxious arousal and avoidance, even after adjusting for previously identified risk factors for stroke-induced PTSD. To our knowledge, this is the first study investigating inhospital psychophysiological response following a medical trauma as a predictor of subsequent symptoms of PTSD.

The findings of the present study are in line with prior research demonstrating that heightened SC reactivity while recalling a nonmedical traumatic event in the days following trauma exposure predicts risk for PTSD symptom development [11]. Additionally, there is some prior evidence that measures of heightened physiological reactivity may be differentially related to PTSD symptom dimensions, as we observed. For example, a study performed in a trauma-exposed civilian sample found that exaggerated fear-potentiated startle (FPS) in response to danger cues predicted reexperiencing symptoms of PTSD [40]. In the current study, we only detected significant associations of SC reactivity with the higher-order fear dimension and the lower-order dimensions of avoidance and anxious arousal. We detected an unadjusted association of small-to-medium effect size between SC reactivity and reexperiencing symptoms in the current study; however, it did not reach the level of statistical significance. Further research in larger samples is needed to determine whether these discrepancies between our results and those of previous research reflect low statistical power in the current investigation or more substantive differences, such as participants' index trauma (medical vs. nonmedical trauma ([5, 41])) and/or psychophysiological measures (SC vs. fear-potentiated startle, which represent distinct neural pathways [42–44]).

There are also potential temporal differences that should be considered when modeling stroke-induced PTSD risk based on psychophysiology. For example, a study exploring psychophysiological predictors of PTSD development in women following an assault that used a similar protocol to the present study (i.e., measuring SC reactivity to recalling a traumatic event) did not find that SC reactivity predicted PTSD symptom development at 3 months posttrauma, although they did find that other physiological indicators of arousal, including heart rate reactivity and recovery, were associated with reexperiencing symptoms of PTSD [45]. However, that study examined SC reactivity during the first month following trauma exposure (*M* = 13.1 days), whereas the present study measured SC reactivity within days of the traumatic event (*M* = 1.69 days). The study by Hinrichs et al. [11] was similarly performed inhospital within a brief period following trauma exposure (*M* = 4.2 hours), potentially indicating that earlier measurement of SC reactivity may capture acute changes in arousal that develop in response to trauma and precede the development of PTSD symptoms.

Our findings suggest the potential utility of SC reactivity measurement during the brief period following a medical or nonmedical trauma for predicting possible vulnerability to the development of fear-related PTSD symptoms. Indeed, we found that the effect size for SC reactivity was of similar magnitude to the effect sizes for the psychosocial predictors of PTSD symptom development (e.g., perceived threat, acute stress symptoms) and larger than the effect sizes for known demographic predictors (e.g., gender, age) examined in this study. Although effect sizes for SC reactivity were slightly attenuated when individuals with probable PTSD due to a prior trauma at baseline were excluded, SC reactivity remained significantly associated with stroke/TIA-induced PTSD symptoms. The tendency to experience greater SC reactivity to recalling a traumatic event could have been a risk factor for both PTSD symptoms due to a prior trauma and for stroke/TIA-induced PTSD symptoms; it is also possible that those with (vs. without) PTSD due to a prior trauma might exhibit greater SC reactivity to recalling the new stroke/TIA traumatic event. Longitudinal research in larger samples is needed to evaluate these hypotheses.

If the present findings are replicated (particularly in larger samples), the results may be useful in guiding future PTSD prevention and intervention models, especially those aimed at reducing fear-related PTSD mechanisms. Machine learning algorithms have recently been shown to predict PTSD symptom development to a high degree of accuracy based on risk measurement obtained while an individual is being treated in the emergency department following a traumatic event [46]. These machine learning algorithms mainly integrate information from self-report questionnaires and extracted from medical records, and they have generally been tested in samples who experienced nonmedical traumas. The results from the current study offer a possible expansion to such algorithms in both kinds of measures that could be considered (i.e., objective measures of autonomic arousal) and the populations in which they could be applied (i.e., individuals who have experienced medical traumas). In addition, of the PTSD interventions that target fear-related mechanisms, prolonged exposure (PE) has the most well-documented efficacy for reducing fear-related symptoms and is considered a first-line treatment for PTSD [47]. It is possible that individuals who demonstrate heightened SC reactivity in the days following a medical trauma may benefit from a form of PE therapy related to their stroke experience. Indeed, there is initial evidence from a pilot study that a modified PE intervention initiated in the emergency department within a brief period following trauma was successful at reducing symptoms of PTSD in individuals with nonmedical trauma [48].

Future directions of this line of research should include longer follow-up of individuals who exhibited heightened SC reactivity to see how this biomarker may relate to PTSD symptom trajectories in the months and years following a stroke. Initial evidence suggests that fear-related PTSD symptom dimensions may have particular relevance for understanding physical health outcomes in individuals with PTSD, and more research is needed to test whether SC reactivity in the acute aftermath of a stroke is related to physical recovery and course of illness following a medical trauma. For example, a study of sudden cardiac arrest survivors found that greater fear-related (i.e., hyperarousal) symptoms of PTSD in response to the cardiac event were related to greater risk for the composite outcome of major adverse cardiovascular event and/or all-cause mortality [16]. Another study found that symptoms of intrusions 1 month following an acute coronary syndrome independently predicted heightened risk of the combined major adverse cardiovascular event and/or all-cause mortality endpoint [49].

Limitations of the present study include the relatively small sample size. Additionally, despite the range of PTSD symptoms that developed in response to stroke/TIA, many individuals in the study reported few to no PTSD symptoms. Thus, these associations should be viewed as preliminary and as proof of concept in need of further confirmation in larger samples. Additionally, the dysphoric arousal model was developed using confirmatory factor analysis of *DSM-IV* PTSD symptom criteria, which consists of 17 symptoms. Although this model has been found to provide a good fit to the 20 PTSD symptoms specified in *DSM-5*, additional, more nuanced models have also been developed for *DSM-5* PTSD [15]. This study also did not examine stroke-related damage to specific brain areas and how that might differentially impact psychophysiological reactivity and/or PTSD symptom development. Research in larger samples is needed that includes targeted identification of damage to brain regions (e.g., emotion processing centers) and its impact on stroke-induced PTSD and risk factors. Nevertheless, this study also contains notable strengths. We utilized a multimethod measurement approach, including subjective (self-report) and objective (data extraction from patients' medical charts, psychophysiological responses) data. Further, the study employed a longitudinal design, allowing us to capture an individual's psychological response to a stroke/TIA event in the acute aftermath of and one month following the event. The mobile SC measurement protocol permitted psychophysiological assessment in a naturalistic, hospital-based setting, thereby increasing the external validity of our findings. Finally, employing a dimensional approach to PTSD symptom measurement allowed for a more nuanced understanding of both predictors of PTSD symptom manifestations and potential mechanisms for the development and intervention of the disorder following a medical trauma.

## 5. Conclusions

In this first study of the association between inhospital psychophysiological reactivity to trauma reminders and subsequent PTSD symptoms in patients following a medical trauma, we demonstrated that greater SC reactivity to recalling a stroke/TIA traumatic event was predictive of higher- and lower-order dimensions of fear-related PTSD symptoms at 1-month postdischarge. Further research is needed to assess whether this biomarker relates to long-term psychological and physical health outcomes and whether individuals who exhibit greater SC reactivity following stroke/TIA may benefit from targeted interventions in the inpatient setting.

## Figures and Tables

**Table 1:** Participant characteristics for individuals who completed the psychophysiology substudy, *n* = 64.

Characteristic	% (*n*)	*M* (SD)	Observed range	Possible range	Valid *n*
*Sociodemographic factors*					
Age, years		61.07 (15.7)	25-87		64
Gender (%)					64
Male	43.8 (28)				
Female	56.3 (36)				
Race/ethnicity (%)					64
Hispanic	42.2 (27)				
Non-Hispanic White	20.3 (13)				
Non-Hispanic Black	29.7 (19)				
Non-Hispanic other	7.8 (5)				
Educational attainment (%)					64
Less than or some HS	18.8 (12)				
HS graduate	20.3 (13)				
Trade school/some college	20.3 (13)				
College graduate	20.3 (13)				
Graduate school	20.3 (13)				
*Medical factors*					
Index event category (%)					64
Stroke	89.1 (57)				
TIA	1.6 (1)				
Other	10.4 (6)				
NIH stroke scale score		3.39 (3.40)	0-20	0-42	56
History of prior stroke/TIA (%)	18.8 (12)				64
Charlson Comorbidity Index		1.26 (1.50)	0-7	0-25	64
*Psychosocial factors*					
Perceived threat during stroke/TIA⁣^∗^		7.73 (6.12)	0-20	0-21	63
Acute posttraumatic stress symptoms⁣^∗^		22.00 (7.54)	14-55	14-70	64
Stroke-induced PTSD symptoms at 1 month					64
Total symptom severity		8.16 (8.68)	0-38	0-80	
Total higher-order fear symptoms		4.27 (4.80)	0-24	0-36	
Anxious arousal		1.70 (1.84)	0-6	0-8	
Avoidance		0.88 (1.37)	0-5	0-8	
Reexperiencing		1.69 (2.81)	0-14	0-20	
Total higher-order dysphoria symptoms		3.89 (4.96)	0-22	0-44	
Dysphoric arousal		1.39 (1.83)	0-6	0-16	
Emotional numbing		2.50 (3.62)	0-16	0-28	
Probable PTSD at 1 month (PCL-5 score ≥ 33)	3.1 (2)				

Note: HS=high school; M=mean; PTSD=posttraumatic stress disorder; SD=standard deviation; TIA=transient ischemic attack. ⁣^∗^Data for these variables were imputed to account for missingness.

**Table 2:** Unadjusted associations between skin conductance reactivity and PTSD symptoms.

	Total symptoms	H/O fear	H/O dysphoria	AA	RE	AV	EN	DA
Correlation (*r*)	.23	.30⁣^∗^	.11	.27⁣^∗^	.22	.25⁣^∗^	.07	.16
*p* value	.070	.016	.393	.035	.087	.043	.586	.215

Note: AA=anxious arousal; AV=avoidance; DA=dysphoric arousal; EN=emotional numbing; H/O=higher order; PTSD=posttraumatic stress disorder; RE=reexperiencing.

**Table 3:** Regression parameters for associations between skin conductance reactivity with higher-order fear, anxious arousal, and avoidance symptom dimensions when adjusting for relevant covariates.

Symptom dimension	*b*	95% CI	*β*	*p* value
*Higher-order fear symptoms*				
Age	0.07	[-0.03, 0.17]	.21	.162
Gender	0.79	[-1.63, 3.20]	.08	.514
Charlson Comorbidity Index	-0.16	[-0.93, 0.61]	-.05	.671
NIH stroke scale	-0.12	[-0.49, 0.26]	-.08	.536
Perceived threat during stroke/TIA	0.20	[-0.01, 0.40]	.25	.061
Acute posttraumatic stress disorder symptoms	0.25	[0.07, 0.43]	.40	.006⁣^∗^
SC reactivity to recalling the stroke/TIA	1.15	[0.32, 1.99]	.36	.008⁣^∗^
*Lower-order anxious arousal symptoms*				
Age	0.03	[-0.01, 0.06]	.22	.112
Gender	0.55	[-0.32, 1.42]	.15	.212
Charlson Comorbidity Index	0.02	[-0.26, 0.29]	.01	.908
NIH stroke scale	0.03	[-0.10, 0.17]	.06	.610
Perceived threat during stroke/TIA	0.12	[0.04, 0.19]	.37	.003⁣^∗^
Acute posttraumatic stress disorder symptoms	0.10	[0.03, 0.16]	.39	.004⁣^∗^
SC reactivity to recalling the stroke/TIA	0.40	[0.09, 0.70]	.32	.011⁣^∗^
*Lower-order avoidance symptoms*				
Age	0.03	[-0.00, 0.06]	.29	.061
Gender	0.35	[-0.35, 1.05]	.13	.323
Charlson Comorbidity Index	0.07	[-0.16, 0.29]	.07	.553
NIH stroke scale	-0.01	[-0.12, 0.10]	-.02	.905
Perceived threat during stroke/TIA	0.02	[-0.04, 0.08]	.10	.459
Acute posttraumatic stress disorder symptoms	0.09	[0.03, 0.14]	.48	.002⁣^∗^
SC reactivity to recalling the stroke/TIA	0.25	[0.00, 0.49]	.27	.047⁣^∗^

Note: CI=confidence interval; SC=skin conductance; TIA=transient ischemic attack. ⁣^∗^*p* value < .05.

## Data Availability

Data is available upon request of the corresponding author (Corinne Meinhausen, cmeinhausen@ucla.edu).

## Supplementary Materials

Supplementary Materials Supplementary Table 1: Cronbach's alpha for the psychosocial covariates with all items answered and the minimum number of items (*k*) that were required to obtain an imputed scale score. Supplementary Table 2: sociodemographic characteristics for participants included in vs. excluded from the analytic sample due to unusable SC data, SC nonresponder status, or missing 1-month PTSD symptom data. Supplementary Table 3: regression parameters for sensitivity analysis of associations between skin conductance reactivity with higher-order fear, anxious arousal, and avoidance symptom dimensions with individuals with probable PTSD in response to a prior trauma at baseline excluded (*n* = 52).
