# Correction to “Skin Conductance Reactivity as a Predictor of Stroke-Induced Posttraumatic Stress Disorder Symptoms: A Dimensional Approach”

**DOI:** 10.1155/da/9859304

**Published:** 2025-06-21

C. Meinhausen, G. J. Sanchez, D. Edmondson, et al., “Skin Conductance Reactivity as a Predictor of Stroke-Induced Posttraumatic Stress Disorder Symptoms: A Dimensional Approach,” *Depression and Anxiety* 2023, no. 1 (2023): 1–12, https://doi.org/10.1155/2023/6671337.

In the article, there are errors in the reported *p*-values found in Paragraph 2 of Subsection 3.2, “In-Hospital SC Reactivity and PTSD Symptoms After Stroke/TIA”. The correct sentence is shown below:

“In fully adjusted models that accounted for age, gender, stroke severity, medical comorbidity, and psychosocial risk factors, SC reactivity remained significantly positively associated with higher-order fear symptoms (*β* = 0.36, *p*=0.008) and lower-order anxious arousal (*β* = 0.32, *p*=0.011) and avoidance symptoms (*β* = 0.27, *p*=0.047; Table 3).”

We apologize for this error.

