# Supplementary material for: Skin Conductance Reactivity as a Predictor of Stroke-Induced Posttraumatic Stress Disorder Symptoms: A Dimensional Approach
Source: Depress Anxiety. 2023 Jul 15;2023:6671337. doi: 10.1155/2023/6671337 (PMC11250708; doi:10.1155/2023/6671337)
Supplement: Supplementary Materials — Supplementary Table 1: Cronbach's alpha for the psychosocial covariates with all items answered and the minimum number of items (k) that were required to obtain an imputed scale score. Supplementary Table 2: sociodemographic characteristics for participants included in vs. excluded from the analytic sample due to unusable SC data, SC nonresponder status, or missing 1-month PTSD symptom data. Supplementary Table 3: regression parameters for sensitivity analysis of associations between skin conductance reactivity with higher-order fear, anxious arousal, and avoidance symptom dimensions with individuals with probable PTSD in response to a prior trauma at baseline excluded (n = 52). [file 6671337.f1.docx]

**Supplementary Table 1. Cronbach’s alpha for the psychosocial covariates with all items answered and the minimum number of items (k) that were required to obtain an imputed scale score.**

| **Psychosocial Covariate** | **α** | **k** |
| --- | --- | --- |
| Perceived threat during stroke/TIA (7 items) | .88 | 3 items |
| Acute posttraumatic stress disorder symptoms (14 items) | .77 | 9 items |

**Supplementary Table 2. Sociodemographic characteristics for participants included in vs. excluded from the analytic sample due to unusable SC data, SC non-responder status, or missing 1-month PTSD symptom data.**

|  | **Included in Analytic Sample (*N*=64)** | **Excluded from Analytic Sample (*N*=34)** |  |  |
| --- | --- | --- | --- | --- |
| **Characteristic** | ***M* (*SD*) or % (*n*)** | ***M* (*SD*) or % (*n*)** | **Test Statistic** | ***p*-value** |
| **Age, years** | 61.1 (15.7) | 59.1 (18.1) | *t*(96) = 0.56 | .576 |
| **Gender, %** |  |  | *X*^2^(98) = 1.00 | .754 |
| Female | 56.3 (36) | 52.9 (18) |  |  |
| Male | 43.8 (28) | 47.1 (16) |  |  |
| **Race/ethnicity, %** |  |  | *X*^2^(98) = 2.00 | .573 |
| Hispanic | 42.2 (27) | 35.3 (12) |  |  |
| Non-Hispanic White | 20.3 (13) | 20.6 (7) |  |  |
| Non-Hispanic Black | 29.7 (19) | 41.2 (14) |  |  |
| Non-Hispanic Other | 7.8 (5) | 2.9 (1) |  |  |
| **Educational attainment, %** |  |  | *X*^2^(98) = 4.34 | .362 |
| Less than or some HS | 18.8 (12) | 29.4 (10) |  |  |
| HS graduate | 20.3 (13) | 23.5 (8) |  |  |
| Trade school/some college | 20.3 (13) | 11.8 (4) |  |  |
| College graduate | 20.3 (13) | 26.5 (9) |  |  |
| Graduate school | 20.3 (13) | 8.8 (3) |  |  |

*Note.* HS=high school; M=mean; SD=standard deviation.

**Supplementary Table 3. Regression parameters for sensitivity analysis of associations between skin conductance reactivity with higher-order fear, anxious arousal, and avoidance symptom dimensions with individuals with probable PTSD in response to a prior trauma at baseline excluded (n=52).**

| **Symptom Dimension** | ***b*** | **95% CI** | **β** | ***p*-value** | |
| --- | --- | --- | --- | --- | --- |
| **Higher-order Fear Symptoms** |  |  |  |  | |
| Age | 0.01 | [0.08, 0.11] | 0.04 | | .793 |
| Gender | 0.36 | [-1.75, 2.47] | 0.05 | | .731 |
| Charlson Comorbidity Index | -0.30 | [-1.03, 0.43] | -0.11 | | .412 |
| NIH Stroke Scale | -0.03 | [-0.35, 0.29] | -0.03 | | .847 |
| Perceived threat during stroke/TIA | 0.10 | [-0.09, 0.29] | 0.15 | | .031* |
| Acute posttraumatic stress disorder symptoms | 0.17 | [0.02, 0.33] | 0.34 | | .298 |
| SC reactivity to recalling the stroke/TIA | 0.94 | [0.22, 1.65] | 0.36 | | .012* |

| **Lower-order Anxious Arousal Symptoms** |  |  |  |  |
| --- | --- | --- | --- | --- |
| Age | 0.02 | [-0.02, 0.06] | 0.17 | .297 |
| Gender | 0.42 | [-0.48, 1.31] | 0.12 | .353 |
| Charlson Comorbidity Index | -0.03 | [-0.34, 0.28] | -0.02 | .851 |
| NIH Stroke Scale | 0.06 | [-0.08, 0.19] | 0.11 | .395 |
| Perceived threat during stroke/TIA | 0.09 | [0.01, 0.17] | 0.30 | .018* |
| Acute posttraumatic stress disorder symptoms | 0.08 | [0.02, 0.15] | 0.37 | .033* |
| SC reactivity to recalling the stroke/TIA | 0.37 | [0.06, 0.67] | 0.33 | .019* |

| **Lower-order Avoidance Symptoms** |  |  |  |  |
| --- | --- | --- | --- | --- |
| Age | 0.00 | [-0.03, 0.03] | 0.00 | .992 |
| Gender | 0.21 | [-0.42, 0.83] | 0.09 | .502 |
| Charlson Comorbidity Index | -0.11 | [-0.32, 0.11] | -0.13 | .317 |
| NIH Stroke Scale | -0.02 | [-0.11, 0.08] | -0.05 | .712 |
| Perceived threat during stroke/TIA | 0.00 | [-0.05, 0.06] | 0.01 | .039* |
| Acute posttraumatic stress disorder symptoms | 0.05 | [0.003, 0.10] | 0.34 | .946 |
| SC reactivity to recalling the stroke/TIA | 0.21 | [0.001, 0.43] | 0.29 | .049* |

*Note*. CI=confidence interval; SC=skin conductance; TIA=transient ischemic attack

**p*-value <.05
